# Acetate: friend or foe? Efficient production of a sweet protein in *Escherichia coli* BL21 using acetate as a carbon source

**DOI:** 10.1186/s12934-015-0299-0

**Published:** 2015-07-25

**Authors:** Serena Leone, Filomena Sannino, Maria Luisa Tutino, Ermenegilda Parrilli, Delia Picone

**Affiliations:** Department of Chemical Sciences, University of Naples Federico II, via Cintia, 80126 Naples, Italy

**Keywords:** Acetate, pH control, Lactose induction, Limiting oxygenation, MNEI

## Abstract

**Background:**

*Escherichia coli* is, to date, the most used microorganism for the production of recombinant proteins and biotechnologically relevant metabolites. High density cell cultures allow efficient biomass and protein yields. However, their main limitation is the accumulation of acetate as a by-product of unbalanced carbon metabolism. Increased concentrations of acetate can inhibit cellular growth and recombinant protein production, and many efforts have been made to overcome this problem. On the other hand, it is known that *E. coli* is able to grow on acetate as the sole carbon source, although this mechanism has never been employed for the production of recombinant proteins.

**Results:**

By optimization of the fermentation parameters, we have been able to develop a new acetate containing medium for the production of a recombinant protein in *E. coli* BL21(DE3). The medium is based on a buffering phosphate system supplemented with 0.5% yeast extract for essential nutrients and sodium acetate as additional carbon source, and it is compatible with lactose induction. We tested these culture conditions for the production of MNEI, a single chain derivative of the sweet plant protein monellin, with potential for food and beverage industries. We noticed that careful oxygenation and pH control were needed for efficient protein production. The expression method was also coupled to a faster and more efficient purification technique, which allowed us to obtain MNEI with a purity higher than 99%.

**Conclusions:**

The method introduced represents a new strategy for the production of MNEI in *E. coli* BL21(DE3) with a simple and convenient process, and offers a new perspective on the capabilities of this microorganism as a biotechnological tool. The conditions employed are potentially scalable to industrial processes and require only low-priced reagents, thus dramatically lowering production costs on both laboratory and industrial scale. The yield of recombinant MNEI in these conditions was the highest to date from *E. coli* cultures, reaching on average ~180 mg/L of culture, versus typical LB/IPTG yields of about 30 mg/L.

**Electronic supplementary material:**

The online version of this article (doi:10.1186/s12934-015-0299-0) contains supplementary material, which is available to authorized users.

## Background

*Escherichia coli* is one of the microorganisms of choice for the production of recombinant proteins at the industrial level. Its use in high density cell cultures allows one to obtain large amounts of unglycosylated, heterologous proteins, with limited production costs and optimized volumetric yields [1, 2]. For this purpose, one of the most common system, at least on the laboratory scale, involves the use of BL21(DE3) cells in combination with the lactose/IPTG inducible pET plasmids (Novagen) [3]. Cells are routinely grown on rich media, such as Luria–Bertani or Terrific Broth [4]. Alternatively, minimal buffered media such as M9, in combination with a wide variety of carbon sources, can be used [5]. In general, defined media are preferred in industrial applications, due to the possibility of easy scale up and careful control of all nutrients concentration [6]. Within the wide panel of possible carbon sources, glucose and glycerol are the most utilized because of their convenience, efficiency and ready availability.

Besides all said advantages, batch cultures of *E. coli* in the presence of excess glucose or glycerol produce acidic fermentation by-products, in particular acetate [7, 8]. Acetate is a known inhibitor of biomass and recombinant protein production [9, 10], and the extent of its production is related to bacterial growth rate and to the availability of the carbon source [11, 12], and is directly involved in the regulation of the central carbon metabolism [13]. At pH 7.0–7.5, acetate is present in equilibrium with undissociated acetic acid. The latter, unlike charged acetate ions, can migrate uncontrolledly through bacterial membranes, disrupting the transmembrane ΔpH and impairing cells viability [14]. For this reason, several techniques have been devised to limit acetate accumulation. These include modifications of the growth medium composition through the addition of amino acids or minerals [15, 16], the design of different process strategies (i.e. fed batch or dialysis culture) [17, 18] or gene engineering on the microorganisms to reduce acetate production and consequent accumulation [10]. These methods are widely reviewed elsewhere [7, 19]. Cultures of *E. coli* K12 tend to produce more acetate compared to BL21 [20, 21]. This is one of the reasons why, although historically adopted in industrial processes, strain K12 is being gradually replaced by BL21 as the preferred microbial host for recombinant protein production. Moreover, recent multi-omics analysis have demonstrated that, compared to strain K12, BL21 possesses superior balance between amino acids production and degradation machineries, thus resulting in more efficient protein yields [22]. Lower acetate production by BL21 compared to K12 is believed to be, in part, also a consequence of a more active glyoxylate shunt, which allows recycling part of the acetate produced during the fermentation toward other gluconeogenic cycles [23, 24]. A recent paper demonstrated that the phenotypic differences between the two strains is due to the high expression of acetyl-CoA synthetase (*acs*) in glucose exponential phase in BL21, which allows the simultaneous consumption of acetate and glucose [25].

Besides being a by-product of fermentative metabolism, acetate and other short chain fatty acids can be used by *E. coli* as carbon sources in conditions of nutrient limitation. Acetate, in its anionic form, cannot diffuse through the membranes, and enters the cells through a transporter-mediated mechanism [26]. Subsequently, it is introduced in the tricarboxylic acid cycle through the glyoxylate shunt, as evidenced by gene profiling [27]. Growing BL21 cells on minimal media with acetate as the sole carbon source proceeds with a longer lag phase and only a slight decrease of the final biomass compared to other gluconeogenic carbon sources in shake flask cultures [28]. Acetate is a quite inexpensive substrate, readily available and well suited for large scale productions. Nonetheless, to our knowledge, no attempt has ever been made to exploit acetate metabolism to express recombinant proteins at high yields.

In the process of defining an optimal condition for large scale manufacturing of the sweet protein MNEI, we tested protein production in a new complex medium containing acetate as a carbon source. The medium was initially based on a pH 7.0 phosphate buffering system containing 0.5% yeast extract as a supplement of essential micronutrients, growth factors and amino acids. A recent paper by Wang et al. [29] has shown that an alkaline shift of the medium pH (pH 7.5–8.5) helps in reducing acetate stress in BL21 when the bacteria are grown on rich media. The authors hypothesized that increasing the medium pH could help reduce the concentration of undissociated, toxic, acetic acid, therefore limiting free diffusion of acetic acid and accumulation of intracellular acetate. We decided to test the effects of pH variations on recombinant protein production when acetate was used as the only supplemental carbon source, assuming that the phenomenon could also be related to its efficient utilization.

When using BL21(DE3)/pET systems, recombinant gene expression is controlled by the T7 promoter and *lac* system. Induction is conventionally achieved with the non-digestible IPTG, although this reagent is extremely expensive and therefore rarely used in industrial scale ups. Moreover, excess IPTG introduces a metabolic burden, and its by-products can ultimately be toxic for the cells [30]. In recent years, methods that replaced IPTG induction with the safer and cheaper lactose or galactose have been developed [31–34]. Here we show that, using our acetate based medium, recombinant protein synthesis can be efficiently induced with 1 mM lactose.

We tested the performance of the new growth medium for the recombinant production of MNEI, an 11 kDa, single chain derivative of monellin, a plant sweet protein [35]. MNEI can interact with the human sweet taste receptor T1R2–T1R3 and humans perceive it as 100,000 times sweeter than glucose on a molar basis [36]. This protein is potentially devoid of adverse effects and is relatively stable at high temperatures, with a melting temperature of 81.6°C at acidic pHs [37]. For all these reasons, MNEI is a likely candidate for the design of new low calorie sweeteners, with potential applications in food and pharmaceutical industries [38]. The possibility of using MNEI as a sugar replacement in industrial preparations relies on the availability of considerable amounts of protein, therefore, much attention has been given to recombinant technologies to obtain large quantities of product, and a variety of host/plasmid combinations have been tested, as reviewed in [39]. Reported protein yields for cultures using the BL21/pET system in rich media are in the range of ~30 mg/L of culture [34, 36, 38]. With the acetate based medium, the production of soluble, functional protein using the same expression system was increased about six folds.

## Results

### Optimization of the acetate based medium for the production of MNEI in shake flasks

**Figure 1:**
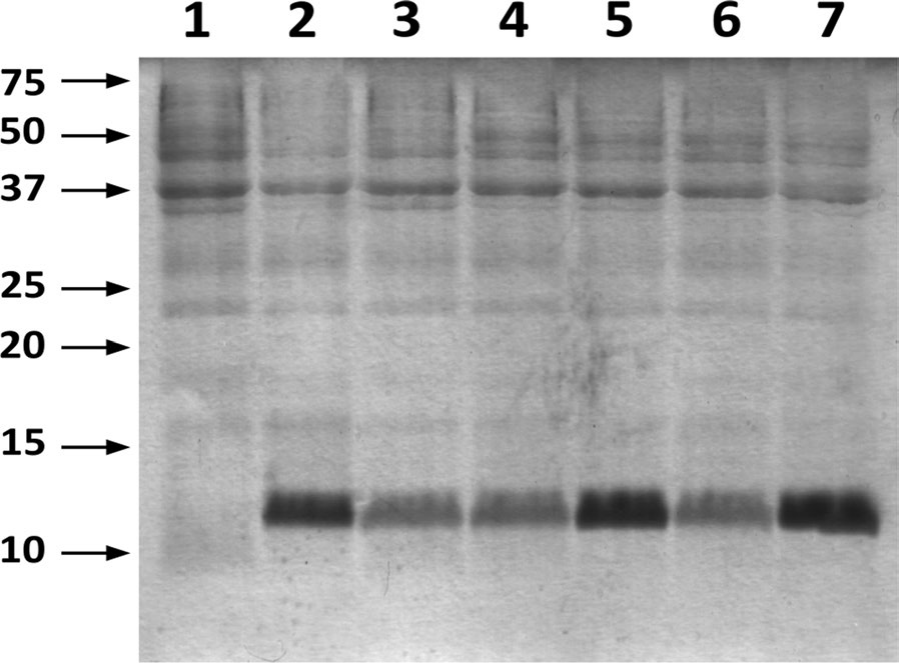
Optimization of lactose concentration for induction in control conditions. Coomassie stained SDS-PAGE profile of the total protein extract from cell cultured in LB and induction with either IPTG or lactose with different post-induction times. 5 μg of soluble protein extract were loaded in each lane. *Lane 1* no induction; *2* 0.4 mM IPTG, 3 h post-induction; *3* 0.4 mM IPTG, 20 h post-induction; *4* 1 mM lactose, 3 h post-induction; *5* 1 mM lactose, 20 h post-induction; *6* 5 mM lactose, 3 h post-induction; *7* 5 mM lactose, 20 h post-induction.

**Figure 2:**
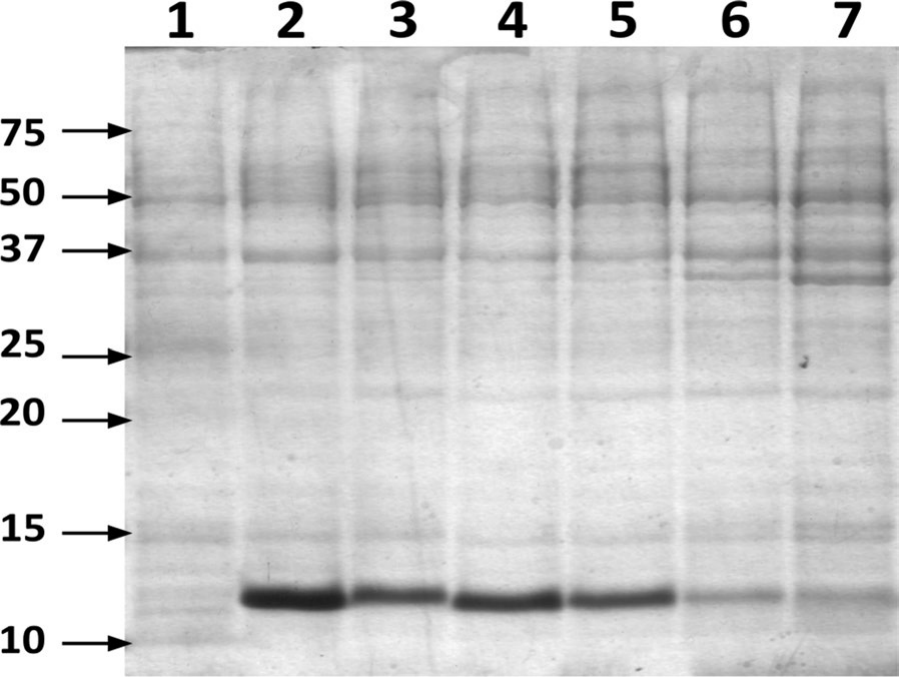
Efficiency of acetate and other carbon sources with lactose induction. When using sodium acetate, the efficiency of lactose as inductor of protein synthesis is superior to other commonly used carbon sources. The Coomassie stained SDS-PAGE analysis shows 3 μg of the total protein extract before induction (*lane 1*) and after 20 h post-induction with 1 mM lactose in LB (*lane 2*) or in PY medium supplemented with 0.4% glycerol (*lane 3*), 0.4% acetate (*lane 4*), 0.2% glycerol + 0.2% acetate (*lane 5*), 0.2% glucose + 0.2% acetate (*lane 6*) or 0.4% fructose (*lane 7*).

In order to define a more convenient growth medium with respect to the conditions previously used in the production of MNEI for structural and functional studies [35, 37, 40, 41], we first tested whether lactose could replace IPTG induction in the control (LB) condition. We set up small scale cultures using 250 mL flasks filled with 100 mL of culture media on a rotating shaker at 250 rpm and 37°C. Results from all small scale experiments were compared qualitatively by visual inspection of the Coomassie-stained SDS-PAGE of the total protein extract. We used two different lactose concentrations within the optimal range (1 and 5 mM) [42] and different post-induction times, and found that using 1 mM lactose and prolonging expression to 20 h yielded comparable amounts of recombinant proteins in LB as IPTG induction (Figure 1). This condition was used as the standard induction procedure in all subsequent experiments. The next step was the definition of the new medium, which was initially based on a pH 7.0 phosphate buffer containing 0.25% NaCl and 0.5% yeast extract as a source of amino acids, growth factors and minerals (PY medium). This medium was supplemented with an additional carbon source to a final concentration of 0.4% and protein production was compared after 20 h of 1 mM lactose induction. We evaluated the efficiency of MNEI production when glycerol, acetate, fructose or mixtures of acetate plus glycerol or glucose were present. Total proteins were extracted and analyzed by SDS-PAGE, and all results were also compared to lactose induced expression in LB (Figure 2). Supplementation of PY medium with acetate yielded the best protein expression levels, and the efficiency of protein production in this condition was higher than with conventional carbon sources such as glycerol or fructose. Using mixtures of acetate with glycerol or glucose also decreased the performance compared to the media containing only acetate, probably due to catabolite repression [4, 43].

**Figure 3:**
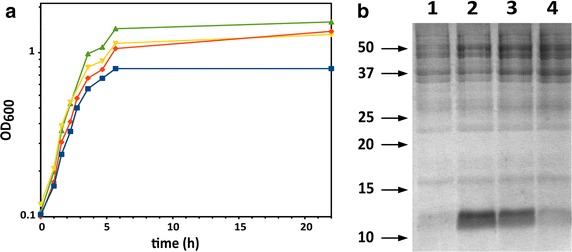
Effect of the medium composition and oxygenation on recombinant protein yield. The figure shows the growth curves (**a**) and Coomassie stained SDS-PAGE of the total protein extract (**b**) after small scale cultures in medium containing 0.25% yeast extract, 0.4% acetate, low oxygenation (*blue curve*, *lane 1*); 0.5% yeast extract, 1% acetate, low oxygenation (*red curve*, *lane 2*); 0.5% yeast extract, 0.4% acetate, low oxygenation (*yellow curve*, *3*); 0.5% yeast extract, 0.4% acetate, high oxygenation (*green curve*, *lane 4*). Induction of protein synthesis was achieved with 1 mM lactose at 0.6 OD_600_. 5 μg of proteins were loaded in each lane. It is visible how only in the presence of sufficient amounts of yeast extract the growth can be sustained. When enough yeast extract is present, the protein production increases with increasing acetate concentration, whereas it drops when more efficient vessel aeration is applied. See the main text for the description of the high and low oxygenation condition.

Having confirmed the possibility of obtaining recombinant protein with the PY-acetate (PYA) medium, we then tried to vary different growth parameters to check whether the efficiency of the protein synthesis could be improved. Our standard shake flask filling (100 mL in 250 mL flasks) was set as the low oxygenation condition, whereas higher oxygenation was achieved by reducing the flask filling level to 50 mL. We also checked the effect of yeast extract reduction (to 0.25%) or sodium acetate increase (to 1%) on biomass and protein production levels. The results of these experiments are reported in Figure 3, which presents the growth curves (panel A) and Coomassie stained SDS-PAGE analysis of the total protein extracts obtained in these conditions (panel B). Reduction of the yeast extract concentration resulted in a sensible biomass and protein yield decrease, as expected since it is known that yeast extract supplementation to culture media is necessary to trigger acetate reduction by *E. coli* [44]. Thus, cultures in these conditions would experience lower nutrients availability, both from the diminution of the yeast extract and from the impaired ability to efficiently utilize the acetate as carbon source. On the other hand, better oxygenation resulted in slightly higher biomass accumulation, but this increase in cell density did not correspond to effective protein production, leading instead to a drop in protein synthesis, as visible in the SDS-PAGE analysis (Figure 3b). Limiting the oxygenation seems therefore required to obtain higher protein yields. In these conditions, and when 0.5% yeast extract was supplied, increasing the concentration of sodium acetate from 0.4 to 1% led to a slight gain in recombinant protein production, while leaving substantially unaffected biomass production. Further experiments with increasing concentrations of sodium acetate in the medium, from 0.4 to 2.5%, were performed, but neither biomass nor protein yield showed significant increase. These results are reported in the supplementary data (Additional file 1: Figure S1).

Recent studies by Wang et al. on BL21 tolerance to acetate [29] have shown that an alkaline shift of the medium pH helps preventing acetate stress on cell growth and protein production in cultures in rich medium. The authors suggested that increasing the medium pH could help retaining the optimal ΔpH between the cells and the surrounding medium, controlling acetate internalization and consequently improving cells viability. We decided to test if a pH shift could be beneficial also in the case of growth on acetate. We started from the PYA medium and adjusted its pH to obtain media with starting pH 7.5, 8.0 and 8.5. These conditions were compared with the PYA medium at pH 7.0 used in the previous experiment. Small scale experiments in these conditions did not evidence significant differences in protein production, although a slight decrease in cell density was observed for the medium at pH 7.0 compared to the other conditions (Additional file 2: Figure S2). From these results, it was apparent that a slight alkaline shift favored cell growth, and that a pH in the range 7.5–8.5 could more efficiently sustain biomass and protein production in the PYA medium.

**Figure 4:**
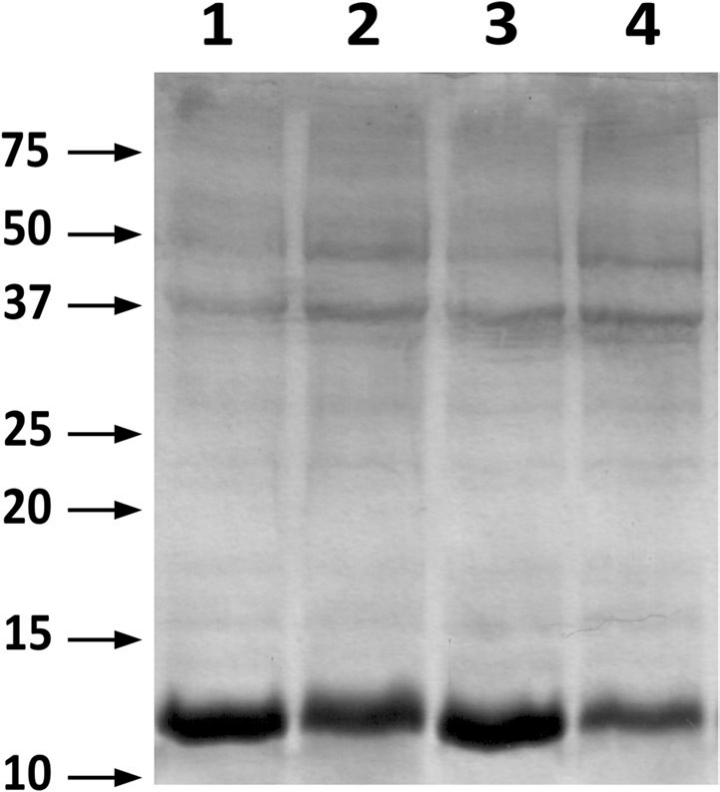
Effect of lactose concentration on protein expression in PYA8 medium. Coomassie stained SDS-PAGE of the total protein extract after small scale cultures in PYA8 medium with different lactose concentrations. 5 μg of soluble protein extract were loaded in each lane. *Lane 1* 1 mM lactose; *lane 2* 5 mM lactose; *lane 3* 1 mM lactose + 1 mM lactose after 3 h; *lane 4* 5 mM lactose + 5 mM lactose after 3 h. Induction was started at 0.6 OD_600_ and continued for 20 h post induction. Using 1 mM lactose provided the best yield of recombinant protein over total protein.

Once this optimal growth condition had been defined, we tried to optimize lactose induction to maximize protein synthesis. We performed the experiments on PYA with a starting pH of 8.0 (PYA8), which is in the middle of the favorable pH conditions explored, and checked induction with 1 or 5 mM lactose. We also explored the effect of a second lactose pulse 3 h after the first induction. The results of these experiments are documented in Figure 4. Although it had been reported that, within the optimal 1–10 mM range, protein expression is proportional to lactose concentration in autoinducing rich media [42], from our experiment it appeared that increasing lactose concentration to 5 mM led to a drop in recombinant protein production, whereas little difference was observed in biomass production in either condition analyzed (data not shown). The administration of one or two 1 mM lactose pulses gave comparable protein yields per total protein, and we decided to maintain the single pulse induction as our standard procedure in larger scale experiments.

### Optimization of the downstream purification procedure

As we were looking to improve the overall process for larger scale production of MNEI, we also perfected the purification procedure. The typical strategy included two chromatographic steps, a cation exchange with a linear gradient of NaCl, followed by a size exclusion chromatography on a Sephadex G-75 column [34, 36]. This latter step is particularly time consuming and difficult to implement on large samples, as size exclusion resins are extremely sensitive to the load volumes and flow rates employed. We developed a one-step ion exchange procedure consisting in a coupled anion/cation exchange purification. The introduction of the preliminary anion exchange chromatography step, performed in a slightly acidic buffer with low ionic strength, allows for a first, rough clean up, because the proteins with acidic and neutral isoelectric points are captured on the column, together with the other negatively charged molecules. Thus, the flow through that reaches the cation resin has already undergone a coarse purification, leading to the removal of many aspecific and secondary interactions, which in turn allows for MNEI to be recovered at high purity from the cation exchanger with a simple step gradient elution, instead of the linear anionic strength gradient used in the previous purification protocol [34, 36]. The ion exchange purification is followed by a desalting step on a Sephadex G-25 resin, which supports loading volumes up to 25% of the total column volume. By choosing to use only resins that were compatible with fast flow rates, this protocol allowed us to complete the purification from the soluble protein extract in just a few hours, and can be further sped and scaled up for larger processes. Once purified, protein was quantified by UV absorbance at 280 nm using an Abs 0.1% of 1.413, as calculated by ProtParam [45] Purity was assessed by Coomassie stained SDS-PAGE and was always higher than 99.5%.

### Process scale up in automatic STR fermenter

To investigate the industrial exploitability of the newly formulated system for the production of MNEI and to better grasp the influence of the different process parameters on protein production in the presence of acetate, we scaled up some of the most representative conditions used in small scale experiments to a 3 L fermentation equipment. In particular, we tested the efficiency of MNEI production in PYA medium with different oxygenation settings (pO_2_ >20% and pO_2_ >10%), different starting pHs (7.0 or 8.0) and increasing acetate concentration (0.4 and 1%) (Fermenter Run, FR 1-4, Table 1). After each run, MNEI was purified and quantified according to the above procedure. The yields reported refer to the final protein recovery.

**Table 1:** Summary of the conditions used in fermenter runs (FR)

	FR1	FR2	FR3	FR4
Sodium acetate	0.4%	0.4%	0.4%	1%
Starting pH	7.0	7.0	8.0	8.0
pO_2_	20%	10%	10%	10%
pH control	No	No	Yes	Yes
Maximum OD_600_/mL	1.3 ± 0.1	1.9 ± 0.1	3.2 ± 0.1	5.0 ± 0.3
μ_max_ (h^−1^)	0.7	0.9	1.0	1.0
DCW (g/L)	0.7 ± 0.1	1.0 ± 0.1	1.7 ± 0.1	2.7 ± 0.2

**Figure 5:**
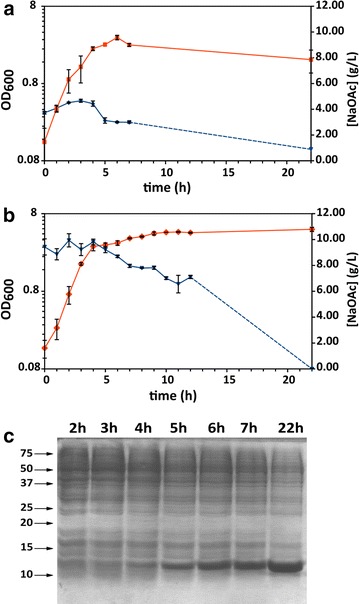
Acetate consumption and temporal evolution of recombinant protein expression in PYA8 medium. Growth curve (*red*) and acetate consumption profile (*blue*) from FR3 (**a**) and FR4 (**b**) and time course evolution of recombinant protein production from FR4 (**c**). From the Coomassie stained SDS-PAGE analysis of the total protein fraction (5 μg) at different during the fermentation (induction at 0.6 OD_600,_ ~1.5 h), it is evident how the majority of protein expression occurs during the stationary phase, when the most part of the acetate in the medium is consumed.

**Figure 6:**
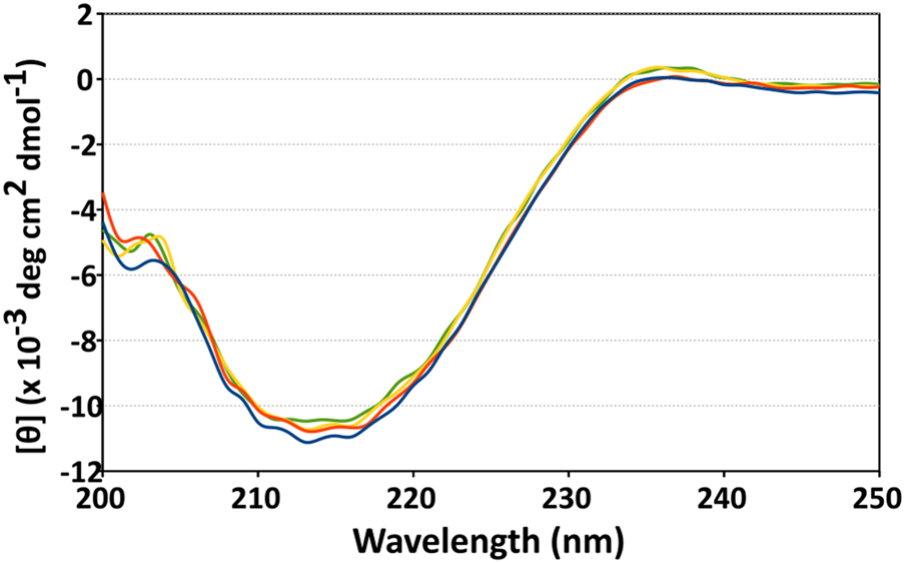
Comparison of the CD spectra of the proteins from fermentation in PYA8 and in LB. Mean residue ellipticity of MNEI obtained from representative fermenter runs (FR2, *blue curve*; FR3, *red curve*; FR4, *yellow curve*; LB control expression, *green curve*). CD spectra are indicative of the typical fold of MNEI, characterized by abundance of β-sheet secondary structures. The spectra do not show any significant difference when the protein is purified from cultures in acetate containing medium or LB.

While performing cell cultures in PYA medium (FR1-2), we noticed that the pH tended to increase over the course of the run, reaching values close to 9. This phenomenon had been previously reported for cultures in rich medium [46, 47]. After depletion of the available carbon sources, amino acids from yeast extract are used by the cells as additional carbon sources with a deamination mechanism that frees ammonia and/or amines in the medium [46, 47]. When the buffering capacity of the phosphate is exhausted, the medium pH rises, reaching unphysiological values (up to 9.0) that could in principle impair biomass production. Nonetheless, we observed protein production after each run. When growing the cells in standard PYA medium (0.4% NaOAc, pH 7.0 uncontrolled, pO_2_ >20%, FR1) we recovered only 14.0 mg of MNEI from 1 L culture. This number increased dramatically when oxygenation was reduced (pO_2_ >10%, FR2), yielding 57.6 mg protein/L culture. Biomass production in the two runs reached 1.3 and 1.9 OD_600_/mL, respectively. When the alkaline shift was applied to the conditions used in FR2 and pH was controlled at 8.0 throughout the fermentation with the dynamic addition of acid (FR3) we noticed major improvements, mostly visible in the increment in biomass production, with a peak of 3.3 OD_600_/mL at the end of the exponential phase. However, after 20 h post induction, cells had entered the death phase and the biomass decayed, leading to non-representative biomass and protein recovery at the end of FR3. Analysis of the acetate consumption profile for this fermentation run showed that acetate was not consumed in the initial phases of the growth, during which its concentration actually increased (Figure 5a), leading to a momentary decrease in the pH. This suggests that the fast increase in biomass in the exponential phase is due to the consumption of other high efficiency carbon sources in the yeast extract. We observed a very high specific growth rate (~1.0 h^−1^), which is consistent with the additional acetate production [48] After about 3 h, acetate concentration reached a peak at 4.6 g/L (6.4 g/L as NaOAc) and then decreased during the whole stationary phase. When starting from a medium containing 1% sodium acetate (FR4), the temporal consumption profile appeared similar to what observed in FR3. Acetate concentration rose slightly in the first phase of the exponential growth and its consumption started only towards the end of the exponential phase, continuing throughout what could be considered a *pseudo*-stationary phase and until completion (Figure 5b). A comparison between the evolution of pH and pO_2_ in the four conditions analyzed is reported in the supplementary data (Additional file 3: Figure S3). The increased availability of acetate led to a noticeable gain in cell density, which reached 5.0 ± 0.3 OD_600_/mL (2.7 ± 0.2 g/L DCW) at the end of the growth (Figure 5b). Soluble protein is efficiently expressed and accumulated throughout the late exponential and the *pseudo*-stationary phase, during which acetate is consumed, as showed in the temporal SDS-PAGE profile after lactose induction (Figure 5c). Recovery after purification was 177 ± 17 mg of protein from 1 L culture (Y_X/S_ = 0.39 ± 0.01 g/g; Y_P/S_ = 0.018 ± 0.002 g/g; Y_P/X_ = 0.07 ± 0.01 g/g. Yields are calculated respect to acetate, MW = 59). The protein fractions obtained from FR2-4 were analyzed by means of circular dichroism (CD) spectroscopy to verify the fold integrity. We found that, for each condition analyzed, the fold was consistent with the protein obtained from the typical LB/IPTG production, as testified by the overlap of the mean residue ellipticity plots (Figure 6).

## Discussion

The accumulation of acetate as a by-product of *E. coli* carbon metabolism has been traditionally regarded as a major limitation to the biotechnological potential of this microorganism [7]. Despite the ascertained ability of growing on acetate as the sole carbon source, to our knowledge no attempt had been done so far to couple acetate metabolism to recombinant protein production. In our study, we have explored this possibility by setting up optimal culture conditions to obtain comparable protein yields to expression in rich media. We used this system to produce MNEI, a recombinant protein of industrial interest, which could potentially find application in the food and beverage industry as a sugar replacer [38]. The interest about this protein has been evidenced by several previous efforts to obtain it at high yields with recombinant methods [39]. We chose to employ cells of *E. coli* BL21(DE3) transformed with the pET22b + _MNEI plasmid. *E. coli* BL21 has widespread utilization in laboratory scale processes, and is gaining increasing attention also in industrial scale processes. Part of the success of this strain is due to its high acetate tolerance, that allows efficient utilization in high density cell cultures with controlled feeding. Increased acetate tolerance is in turn due to an optimal acetate utilization machinery [22, 23]. We found that acetate metabolism can be employed to efficiently produce the recombinant protein in *E. coli* BL21. The choice of the BL21/pET combination also allows the use lactose induction, which is to prefer, due to reduced cost and higher biocompatibility, to conventional IPTG induction [30]. We noticed that careful control has to be applied mostly on two parameters: oxygenation and pH. In experiments with glycerol based autoinducing media, fed batch and glucose fed batch like cultures [47, 49, 50], it had already been observed that protein expression under lactose induction is more efficient in limiting oxygen concentrations. This is also the case in the conditions explored in our study, since protein expression was completely inefficient when using higher oxygenation conditions in both small scale experiments and fermenter productions. The other parameter primarily affecting protein synthesis was the medium pH. It had been already reported that, when a primary carbon source is available, an alkaline shift could improve biomass and recombinant protein production in BL21 in the presence of acetate [29]. Our study shows that increasing the medium pH favors acetate metabolism, sustaining higher cell density in the absence of additional carbon sources. In these conditions, a concentration of sodium acetate in the medium as high as 1% (120 mM) could be consumed to completion during the *pseudo*-stationary growth that follows the exponential phase, with concomitant production of biomass and recombinant protein. This metabolism is likely coupled to the utilization of the amino acids present in the yeast extract as additional carbon sources. Use of amino acids as carbon sources proceeds through deamination and results in net ammonia and amines production, which in turn determines a pH increase [46, 47]. This trend was indeed observed in the fermentations with no pH control (FR1-2), and led to final pH values close to nine. These non-physiological conditions are likely to stop cell growth, inducing partial death and lysis, with consequent decrease of the final biomass and protein yields. By introducing pH control to 8.0, cell death is retarded and biomass accumulation continues throughout the fermentation run, leading to higher cell density and superior protein accumulation, as evidenced in FR3-4. Acetate consumption in these conditions mostly occurs during the *pseudo*-stationary phase, when the majority of the protein accumulation is observed. This system can efficiently be coupled with lactose induction. In general, lactose has been used following primary induction with IPTG [32] or in autoinducing media in the presence of a repressor of the T7*lac* promoter, such as glucose. The pioneering work by Studier on auto-inducing media [49] proved that induction by lactose can be achieved with as little as 0.005%, but is inhibited by certain sugars (primarily glucose) or amino acids. Later studies defined an optimal lactose concentration between 1 and 10 mM to be used for expression of recombinant proteins in *E. coli* BL21 in typical autoinducing media, where additional digestible carbohydrates are normally present [42]. We found that, when using PYA8 medium, optimal induction was obtained with as little as 1 mM lactose, whereas a higher concentration led to a reduction in the expression levels.

## Conclusions

In the present study, we have developed a new strategy for the production of MNEI, a sweet protein with potential application in the field of low calorie sweeteners, obtaining higher protein titers compared to published methods employing the same expression system [39]. We have used *E. coli* BL21(DE3) as the expression host and optimized a medium containing acetate as a carbon source (PYA8) and lactose as inducer. Average protein yield with this method reached 177 mg of protein per liter of culture, which represents a sixfold increase compared to the ~30 mg/L typically obtained from the LB/IPTG protocol [34–36]. The strategy presents obvious advantages over conventional approaches, mostly linked to the affordability and availability of the reagents, and seems promising for potential scale-ups. The purification protocol of MNEI has also been redesigned and it is now suitable for applications on larger production scales. Further validation of this production system will come from the expression of other recombinant proteins with the BL21/pET combination. The batch method here presented is also likely susceptible of further improvements, with the application, for instance, of modified feed strategies, which will certainly be the object of future studies.

## Methods

### Growth conditions

All experiments were performed on *E. coli* BL21(DE3) cells freshly transformed with the pET22b + plasmid (Novagen) carrying the gene coding for MNEI as used in [35]. The gene had been cloned between the *Nde*I and *BamH*I restriction sites of the vector, thus removing the pelB leader sequence and allowing for cytoplasmic protein production. All media contained 100 mg/L Ampicillin for plasmid preservation. For small scale cultures, cells were grown on LB-Agar plates and individual colonies were inoculated in 10 mL LB (10 g/L Tryptone, 10 g/L NaCl, 5 g/L Yeast-Extract) and grown over night at 37°C on a rotating shaker at 250 rpm. Pre-cultures were then diluted at 0.1OD_600_ in 100 mL of the final culture medium in 250 mL flasks and incubated on a rotating shaker at 250 rpm and 37°C. Cell growth was monitored by OD_600_ readings and protein synthesis was induced at 0.6 OD_600_ and continued for 20 h unless otherwise specified. For fermenter runs, cells from agar plates were inoculated in 5 mL LB and growth for 6 h on a rotating shaker at 250 rpm. This starting cultures were diluted 1/100 in 100 mL of the different media, grown in 250 mL flasks at 37°C for 20 h and used to inoculate the culture in fermenter. Fermentations were carried out in a STR 3L Bioreactor connected to an ADI1030 Bio Controller (Applikon) in the final culture media (1 L working volume) and supplemented with ampicillin 100 mg/L for plasmid selection. The culture was maintained in aerobic conditions (DOT ≥10% unless otherwise specified) by an airflow of 60 L/h and a stirring rate of 250 rpm. When indicated, the culture pH was maintained at 8.00 by automatic addition of H_2_SO_4_ 5% v/v.

The following media were used in the different experiments:

PY medium (12.8 g/L Na_2_HPO_4_·7H_2_O, 3.0 g/L KH_2_PO_4_, 0.5 g/L NaCl, 5.0 g/L yeast extract), PY8 (16.1 g/L Na_2_HPO_4_·7H_2_O, 1.36 g/L KH_2_PO_4_, 0.5 g/L NaCl, 5.0 g/L yeast extract). Sodium acetate (A) was added from a sterile 10× stock solution to the final concentrations indicated in the main text.

Further pH adjustments were made with 0.1 M NaOH or 5% H_2_SO_4_.

Induction of protein expression was realized with 1 mM lactose at 0.6 OD_600_, unless differently specified in the text.

The concentration of acetate in the medium was determined spectrophotometrically [51]. Prior to spectrophotometric analyses, all samples were heated to 80°C for 20′ to denature proteins and were analyzed with the K-ACETRM acetic acid determination kit (Megazyme). Acetate concentration was expressed as sodium salt equivalents.

Protein concentration for electrophoretic analysis was determined by Bradford Assay (Bio-Rad).

### Protein purification

After growth, cells were harvested by centrifugation (4,000×*g*, 4°C, 10′), washed with cold PBS and resuspended in 50 mM NaOAc, pH 5.5 to 50 OD_600_/mL. Cells were disrupted by intermittent sonication on ice (20′) and debris were removed by centrifugation (12,000×*g,* 4°C, 30′). The cell lysate was applied to an anion exchange DEAE-Sepharose (20 mL, GE Lifesciences) column connected in series to a Macro-prep High-S cation exchange column (15 mL, Bio-Rad), equilibrated in the same lysis buffer. The chromatography was monitored by UV absorption at 280 nm. After loading and washing to baseline, the columns were disconnected and MNEI was eluted from the Macro-prep High-S column with 50 mM NaOAc buffer containing 100 mM NaCl. The fractions containing MNEI as assessed by SDS-PAGE were pooled and desalted on a Sephadex G-25 column in 50 mM AcOH and lyophilized. All the resins utilized in the protein purification steps allow fast flows, and a 5 mL/min flow was used in all purification steps. MNEI purity was estimated by analysis of the SDS-PAGE with the Image Lab 5.2 software (Bio-Rad).

### Circular dichroism

Protein fold integrity was assessed by circular dichroism (CD) spectra recorded on a Jasco J-715 spectropolarimeter. Molar ellipticity per mean residue [θ] in deg cm^2^ dmol^−1^ was calculated from the equation: [θ] = [θ]_obs_ mrw/(10 × l × C), where [θ]_obs_ is the ellipticity measured in degrees, mrw is the mean residue molecular weight of the protein (Da), C is the protein concentration in g/mL and l is the optical path length of the cell in cm. Cells of 0.1 cm path length were used. CD spectra were recorded with a time constant of 4 s, a 2 nm band width and a scan rate of 20 nm/min, and the signal was averaged over three scans and baseline corrected by subtracting a buffer spectrum. All spectra were recorded in 20 mM phosphate buffer, pH 2.5. A concentration of 0.25 mg/mL protein was used for each sample, as accurately determined by UV absorbance at 280 nm prior to CD measurement.

## Additional files

Additional file 1:
**Figure S1.** Effect of sodium acetate concentration on biomass and recombinant protein yield. Growth curves (A) and SDS-PAGE (B) of the soluble protein extract after small scale cultures in PY medium containing different sodium acetate concentration: 0.4% (blue curve, lane 1); 1% (red curve, lane 2); 1.5% (yellow curve, lane 3); 2.0% (green curve, lane 4) and 2.5% (dark red curve, lane 5). 3 μg of soluble protein extract were loaded in each lane. It appears that increasing acetate concentration in the medium does not affect the efficiency of protein expression, whereas, without pH or pO_2_ control, biomass tends to decrease with higher acetate concentrations. Additional file 2:
**Figure S2.** Effect of the medium pH on biomass and recombinant protein yield. Growth curves (A) and SDS-PAGE (B) of the soluble protein extract after small scale cultures in PYA medium varying the starting pH. Starting pH of 7.0 (blue curve, lane 1); 7.5 (red curve, lane 2); 8.0 (yellow curve, lane 3); 8.5 (green curve, lane 4). 5 μg of soluble protein extract were loaded in each lane. In small scale experiments, variations of the starting pH above 7.5 did not result in significant changes in either biomass or protein production. A slight decrease in cell density was visible at the end of the culture starting at pH 7.0, but this effect was more significant in fermenter runs. Additional file 3:
**Figure S3.** Biomass, pO_2_ and pH evolution in typical fermenter runs. Growth curves (green), pO_2_ (red) and pH profiles relative to FR1 (panel A), FR2 (panel B), FR3 (panel C) and FR4 (panel D). In the fermentations without pH control, it is evident a drift to high pHs throughout the growth. When alkaline shift and pH control are applied (C-D), an initial slight decrease of the pH is observed, concomitant with acetate production.

## Abbreviations

IPTG: Isopropyl-β-d-1-thiogalactopyranoside
LB: Luria Broth
PYA: phosphate/yeast extract/acetate medium
PYA8: phosphate/yeast extract/acetate pH 8.0 medium
CD: circular dichroism
NaOAc: sodium acetate
DCW: dry cell weight
Y_X/S_: yield of biomass from substrate
Y_P/S_: yield of protein from substrate
Y_P/X_: yield of protein from biomass
