# Supplementary figures and images for: Acetate: friend or foe? Efficient production of a sweet protein in Escherichia coli BL21 using acetate as a carbon source
Source: Microb Cell Fact. 2015 Jul 25;14:106. doi: 10.1186/s12934-015-0299-0 (PMC4514960; doi:10.1186/s12934-015-0299-0)

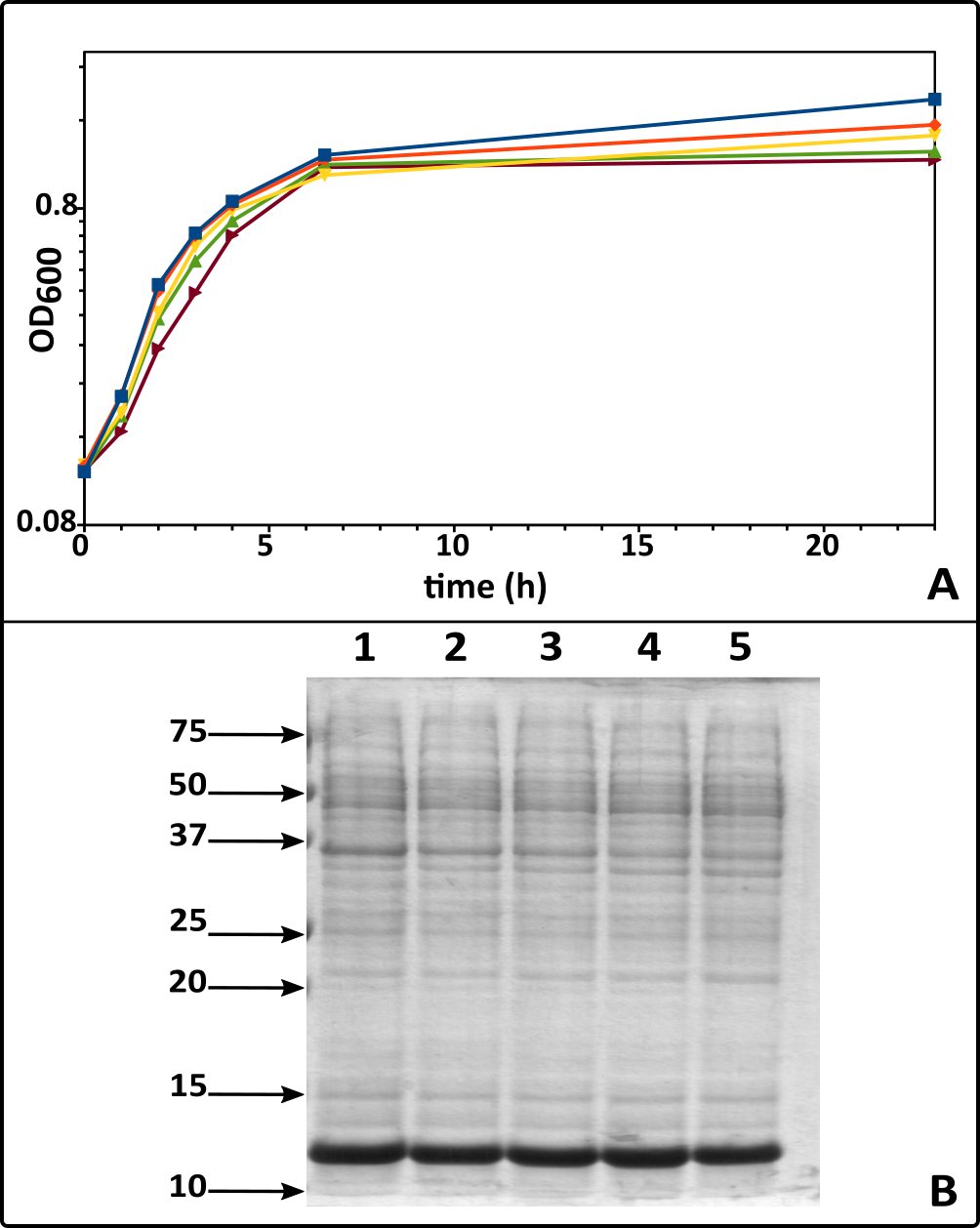

Supplement: Additional file 1: — Figure S1. Effect of sodium acetate concentration on biomass and recombinant protein yield. Growth curves (A) and SDS-PAGE (B) of the soluble protein extract after small scale cultures in PY medium containing different sodium acetate concentration: 0.4% (blue curve, lane 1); 1% (red curve, lane 2); 1.5% (yellow curve, lane 3); 2.0% (green curve, lane 4) and 2.5% (dark red curve, lane 5). 3 μg of soluble protein extract were loaded in each lane. It appears that increasing acetate concentration in the medium does not affect the efficiency of protein expression, whereas, without pH or pO2 control, biomass tends to decrease with higher acetate concentrations. [file 12934_2015_299_MOESM1_ESM.png]

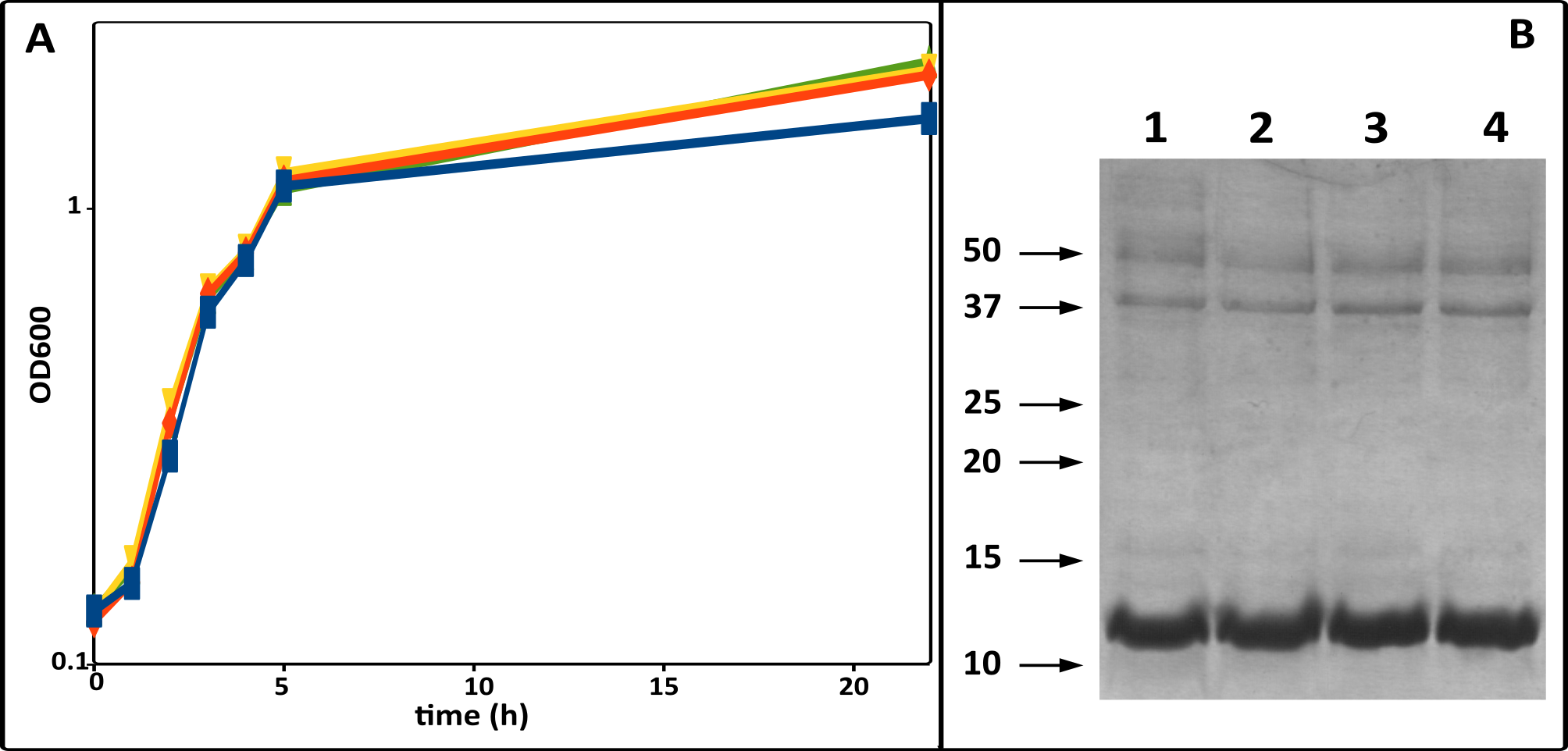

Supplement: Additional file 2: — Figure S2. Effect of the medium pH on biomass and recombinant protein yield. Growth curves (A) and SDS-PAGE (B) of the soluble protein extract after small scale cultures in PYA medium varying the starting pH. Starting pH of 7.0 (blue curve, lane 1); 7.5 (red curve, lane 2); 8.0 (yellow curve, lane 3); 8.5 (green curve, lane 4). 5 μg of soluble protein extract were loaded in each lane. In small scale experiments, variations of the starting pH above 7.5 did not result in significant changes in either biomass or protein production. A slight decrease in cell density was visible at the end of the culture starting at pH 7.0, but this effect was more significant in fermenter runs. [file 12934_2015_299_MOESM2_ESM.png]

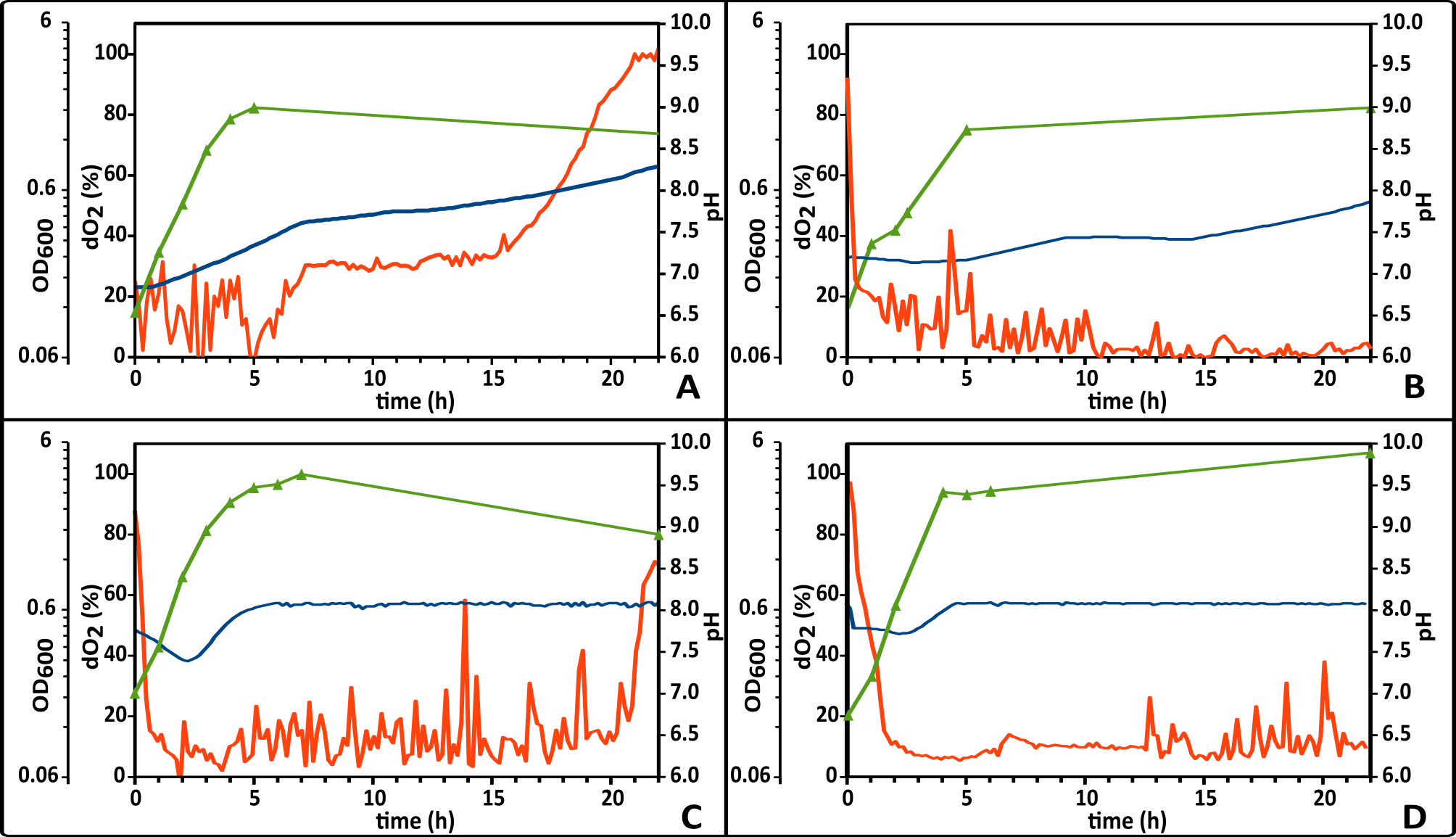

Supplement: Additional file 3: — Figure S3. Biomass, pO2 and pH evolution in typical fermenter runs. Growth curves (green), pO2 (red) and pH profiles relative to FR1 (panel A), FR2 (panel B), FR3 (panel C) and FR4 (panel D). In the fermentations without pH control, it is evident a drift to high pHs throughout the growth. When alkaline shift and pH control are applied (C-D), an initial slight decrease of the pH is observed, concomitant with acetate production. [file 12934_2015_299_MOESM3_ESM.png]
